# Metallurgical Characterization of Penetration Shape Change in Workpiece Vibration-Assisted Tandem-Pulsed Gas Metal Arc Welding

**DOI:** 10.3390/ma13143096

**Published:** 2020-07-10

**Authors:** Habib Hamed Zargari, Kazuhiro Ito, Tsuyoshi Miwa, Pradeep Kumar Parchuri, Hajime Yamamoto, Abhay Sharma

**Affiliations:** 1 Joining and Welding Research Institute, Osaka University, 11-1 Mihogaoka, Osaka 567-0047, Japan; ito@jwri.osaka-u.ac.jp (K.I.); miwa.tsuyoshi@kobelco.com (T.M.); pradeepkumar.rut@gmail.com (P.K.P.); h.yamamoto@jwri.osaka-u.ac.jp (H.Y.); 2 Department of Mechanical and Aerospace Engineering, Indian Institute of Technology Hyderabad, Sangareddy, Telangana 502285, India; abhay@iith.ac.in; 3 Department of Materials Engineering, Faculty of Engineering Technology, KU Leuven, Campus De Nayer, 2860 Sint-Katelijne Waver, Belgium

**Keywords:** tandem gas metal arc welding, pulsed welding, workpiece vibration, penetration shape, Si segregation, resonance phenomenon

## Abstract

Tandem-pulsed gas metal arc welding (TP-GMAW) simultaneously uses two wire-electrodes to enhance the material deposition rate, leading to the generation of a finger-shaped penetration as one of the arcs penetrates deeper than the other. On the other hand, workpiece vibration is one of the techniques used to control the microstructure of weld metal and a heat-affected zone. It is incidentally found that a specific vibration condition changes the finger-shaped penetration into pan-bottom shaped penetration in the TP-GMAW even though the vibration energy is much lower than the arc energy. Microstructure observation and elemental analysis are carried out for the welds fabricated without vibration and with three kinds of vibration modes, namely sine, random, and shock. The specific sine-mode vibration exhibits pan-bottom. The other modes of vibration in the same welding conditions exhibited invariable finger-shaped penetration. The Si atoms as a tracer distribute uniformly in the sine-mode. However, Si atoms segregate at the bottom of the finger-shaped weld metal with the random-mode and shock-mode workpiece vibrations. The weld pool shape change is prominent at a specific frequency. A resonance phenomenon between the droplet flow pattern and the molten material flow in the weld pool is likely to play a vital role in the change.

## 1. Introduction

Vibration-assisted welding (VAW) is known as a successful replacement of post-weld treatments of arc welds. Many benefits of vibration effect on the performance of the welds have been reported over the past decades. Grain refining, decreasing the number of blowholes, heat-affected zone (HAZ) morphology modification, and reducing residual stresses are advantages that lead to improving mechanical properties in the weld zone [1]. The vibrations in the manufacturing processes are imparted on the tool [2,3,4,5,6] and workpiece [7,8,9,10] that correspond to the electrode and weld-pool vibration in welding. The vibration of the arc due to pulsed current also leads to agitation in the weld pool. The weld pool oscillation behavior in the case of gas tungsten arc welding (GTAW) has been studied extensively [11,12,13,14] and is likely best understood. The oscillations are triggered by applying an external force on the weld pool such as superimposing a high current pulse on the welding current or mechanical disturbance of the workpiece. The natural frequency of the partial penetration GTAW pool depends on its geometry, such as the diameter [15]. However, the natural frequency of the partial penetration weld pool is not affected by pool depth, at least under the experimental conditions with low vibration amplitude [12].

In the case of gas metal arc welding (GMAW), the condition is more complicated due to the irregular movement of the liquid metal generated by the impinging droplets [16]. Moreover, the molten droplet itself oscillates due to variation in the electromagnetic force and the surface tension [14,17]. In short-circuit GMAW, the weld pool oscillation is directly related to the arc re-ignition after the interruption of the liquid bridge [18]. The sudden increase of the arc pressure due to the re-ignition depresses the weld pool. Subsequently, as the welding current decreases to its original value, the weld pool surface is brought into oscillation associated with the surface tension and gravity. Fan et al. [19] observed that weld pool oscillation in globular transfer GMAW could be operated by the impinging droplets. Tsai et al. [20] simulated the comprehensive transport phenomena among the electrode, weld pool, and arc plasma during stationary GMAW of AISI 304 stainless steel with a constant current. They showed that the weld pool was depressed by the impinging droplets and then closed due to the hydrostatic force. A complete cycle of weld pool oscillation could not be observed due to a high impinging frequency (almost 60 Hz).

Several analytical models have been developed for calculating the oscillation frequency of a weld pool and droplets following various approaches [21,22,23,24]. These models demonstrate that for each oscillation mode (symmetry, sloshing, etc.), the oscillation frequency depends on the weld pool geometry and the material properties, especially the surface tension and the density of the liquid metal. The surface tension gradient caused by the temperature distribution in the weld pool leads to mass transfer known as the Marangoni convection. The viscosity of the molten metal influences the role of the Marangoni convection and thereby impacts the flow pattern. The top surface and the penetration shape of the weld are impacted by the combined effect of the viscosity of molten metal and surface tension. All the models agree that an increase in surface tension increases the weld pool oscillation frequency and decreases the density and weld pool size. In relatively small weld pools, the surface tension controls the oscillation frequency, while for large weld pools, gravity dominantly controls [21]. Past observations on weld pool oscillation led to the development of oscillation-based penetration sensing during arc welding for maintaining the desired weld penetration [25].

Tandem-wire pulsed gas metal arc welding (TP-GMAW) is a special arrangement wherein two electrodes are used. The electrodes are oriented along the weld seam. The leading electrode acts on the fresh metal while the trailing electrode acts on the molten pool created by the leading electrode. The deposition rate, thus, increases because of the two electrodes. The arc at either electrode passively heats another electrode that increases the overall melting efficiency [26]. The interaction between the arcs influences the arc stability [27], which directly affects the microstructures and properties [28]. In TP-GMAW, the welding currents at the two electrodes are pulsed with a time lag (phase difference) so that the arcs do not ignite at the same time. The phased pulsed currents limit the arc interaction and improve the arc stability. The pulsation, in specific conditions of tandem welding, produces deeper finger-shaped penetration. The leading arc that acts on cold fresh metal constricts and, thereby, the current density increases, which leads to deeper weld penetration [29]. The trailing arc spreads more to the lateral sides and creates a shallow weld pool. This investigation is motivated by an incidental observation with workpiece vibration. While experimenting with TP-GMAW, the finger-shaped penetration changed to a pan-bottom (flat) penetration, although the vibration energy was noticeably smaller than the TP-GMAW heat input. The present study aims to investigate the shape change phenomenon along with the metallurgical aspects of change in vibration frequency, acceleration, welding speed, and time of workpiece vibrations considering different vibration modes.

## 2. Experimental Procedure

**Figure 1:**
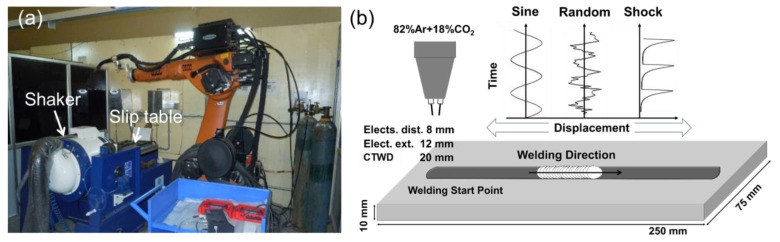
(**a**) A robotic welding instrument and (**b**) a welding set-up used in a vibration assisted welding.

The welding experiments were conducted using a robotic TP-GMAW facility at IIT Hyderabad (Sangareddy, India). The two electrode wires were fed simultaneously through the welding torch and received electric power from two independent power sources, as shown in Figure 1a. The two power sources were synchronized in an anti-phase manner to limit the arc interaction. The robotic welding facility consists of a 6-axis robot (KR 30-3) made by KUKA (Augsburg, Germany), attached to a time-twin digital welding machine from Fronius. The robot had a reach of 2033 mm and a repeatability of +0.06 mm. An electro-dynamic vibration shaker (Sdyn, Roorkee, India) of 1500 kgf capacity was used to generate vibration. The slip table connected to the shaker head was vibrated longitudinally. The fixture was bolted to the slip table with sheets of the insulator and thermal barrier in between. Four wedge clamps were used evenly to fix the workpiece tightly to avoid any kind of distortion during welding. The insulator prevented the flow of current through the slip table and shaker head and thus avoided possible danger to the shaker.

The base metal (BM) used for the investigation was a hot-rolled low carbon steel IS 2062-2011 (equivalent ASTM A1011), consisting of the ferrite-pearlite structure. The material for wire electrode was copper-coated steel designated as ER 70-S made by ADOR (Mumbai, India), with a diameter of 1.2 mm. The chemical composition of the BM and welding wire are listed in Table 1. The ER 70-S is a general-purpose electrode for manual and semiautomatic applications. The selection of the electrode material is an important consideration in this investigation because higher content of Si in ER 70-S compared the base metal acts as the tracing element to visualize the weld pool flow pattern. The average arc currents of the leading and trailing electrodes were set to 180 A. The base and peak current of the pulsed waveform were 100 to 340 A and one pulse one droplet transfer state was maintained at 154 Hz. The two electrodes were kept at 8 mm. A mixed gas of Ar 82%–CO_2_ 18% was used as a shielding gas. The bead-on-plate welds were laid for a length of 200 mm in a flat position, keeping the contact tip to workpiece distance (CTWD) at 20 mm. A schematic illustration of a welding set-up and the vibration modes are shown in Figure 1b.

Three types of vibration mode—sine, shock, and random modes—were considered in this study. The welding speed (*S*) and the parameters of vibration including the vibration frequency (*F*), vibration acceleration (*a*), and the time of vibration after welding ends (*T*) were selected to investigate the vibration effect on penetration shape change. The values of the parameters investigated are listed in Table 2. The vibration displacement (*x_l_*) is defined for the sine mode, for example, in the relation (1):

(1) $$\;x_{l}=\frac{D}{2}\sin\;\left(2\pi Ft\right)$$

where *D* is peak to peak displacement (i.e., *D*/2 is amplitude) and *t* is operation time. The vibration acceleration (*a*) can be obtained from the relation (2):

(2) $$a=-2DF^{2}\pi^{2}\;\sin\;\left(2\pi Ft\right)$$

The vibration acceleration reaches to peak (*G*) when

(3) $$2\pi Ft=\left(2n+1\right)\frac{\pi }{2}\left(n=0,\;1,\;2,\;3,\ldots \right)$$

thus,

(4) $$G=2DF^{2}\pi^{2}$$

Cross-sectional microstructure observations were obtained from specimens produced by the TP-GMAW with (w/) and without (w/o) vibration using optical microscopy (OM, VH-Z100UR, KEYENCE, Osaka, Japan) and scanning electron microscopy (SEM, JSM-6060LV, JEOL, Akishima, Japan). The crystal-orientation and elemental analyses were conducted using SEM equipped, respectively, with an electron back-scatter diffraction (EBSD, NB5000, HITACHI, Chiyoda, Japan) detector and an electron probe microanalyzer (EPMA, JXA-8530F, JEOL, Akishima, Japan), primarily focusing on Si whose content was higher in the filler wire than in the BM. The specimens after polishing were etched by 7 vol% HNO_3_ + 93 vol% CH_3_OH. The Vickers hardness tests were carried out at room temperature with an applied load of 0.98 N and loading time of 30 s at 0.1 mm intervals.

## 3. Results and Discussion

### 3.1. Vibration-Assisted Penetration Shape Change

**Figure 2:**
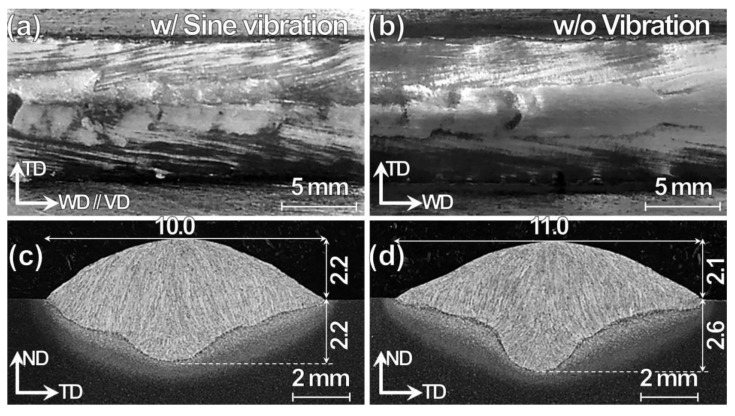
(**a**,**b**) Top-view and (**c**,**d**) cross-sectional optical microscopy (OM) images for Tandem-pulsed gas metal arc welding (TP-GMAW) specimens at 1.2 m/min (**a**,**c**) w/ and (**b**,**d**) w/o sine vibration (vibration frequency (F) = 250 Hz, vibration acceleration peak (G) = 1.2 m/s^2^ and time of vibration after welding ends (T) = 30 s).

Figure 2 shows top-view and cross-sectional OM images of TP-GMAW specimens at 1.2 m/min w/ and w/o sine vibration (F = 250 Hz, G = 1.2 m/s^2^ and T = 30 s). The top-view of bead-on-plate welds exhibit smooth surface without any welding defect such as blowhole for both the specimens (Figure 2a,b). On the other hand, the cross-sectional weld shape is quite different. The weld width and penetration depth are smaller for the specimen w/ vibration, while weld height is not different (Figure 2c,d). The greatest difference between the specimens is the penetration shape: pan-bottom and finger-shaped penetration for the specimen w/ and w/o vibration, respectively. The difference in the shape is likely to be associated with the resonance phenomenon between high-temperature impinging droplets and relatively low-temperature vibrating molten pool. The pan-bottom shaped penetration is also assumed to be caused by molten material flow downwards just before solidification is complete. The vibration amplitude is about 0.5 μm, much smaller than the molten pool size, which can be imagined by the weld width of about 10 mm. The penetration-shape affects HAZ thickness variation below the fusion line. The HAZ (Figure 2c,d) has a light- and dark-gray contrast in the cross-sectional OM images. The HAZ has a relatively uniform thickness from the weld toe to the weld metal (WM) bottom for the specimen w/ vibration (Figure 2c). In contrast, the thickness significantly increases at a concave spot (metallurgical notch) for the specimen’s w/o vibration (Figure 2d). The metallurgical notch occurs at the conjunction of two separate instantaneous weld-pools. The instantaneous weld pools are the results of the varying arcing phenomenon because of the physical separation of the arcs (as in the present case) or instantaneous polarity reversal as in the case of a square sine wave [30]. On the other hand, both the specimens (w/ and w/o) exhibit typical WM and HAZ microstructure of the low carbon steel. The OM images, as shown in Figure 2c,d, exhibit many white curves that indicate grain boundary ferrites formed on former γ grain boundaries. The average grain size of former γ grains seems not to vary with presence or absence of vibration.

**Figure 3:**
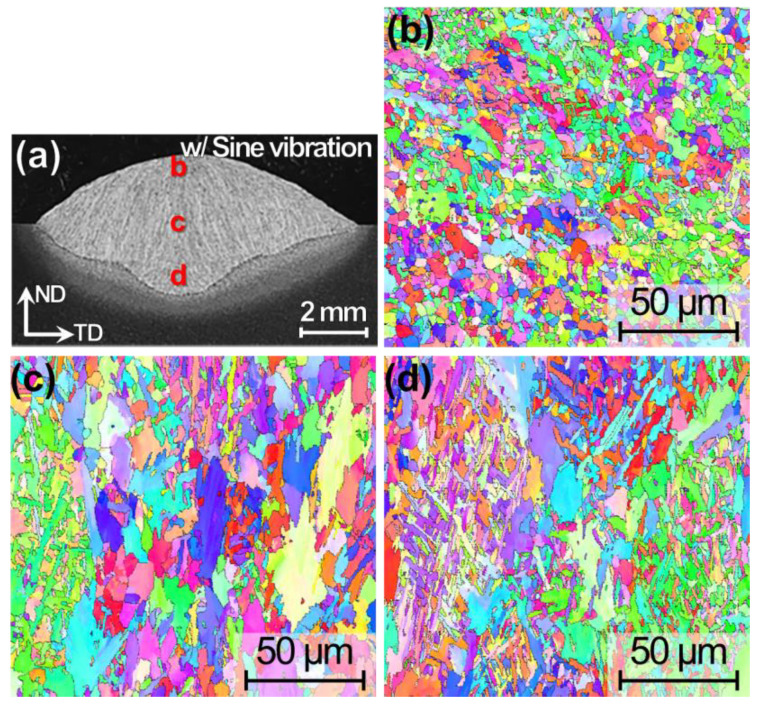
Cross-sectional (**a**) OM image and (**b**–**d**) electron back-scatter diffraction-inverse pole figure (EBSD-IPF) maps for TP-GMAW specimens at 1.2 m/min w/ sine vibration (F = 250 Hz, G = 1.2 m/s^2^ and T = 30 s).

The macroscopic OM images exhibit similar feature in the view point of former γ grains (Figure 2c,d), and thus, for only the specimen w/ vibration, the finer microstructures identified by EBSD-inverse pole figure (IPF) maps are shown in Figure 3. The images are taken from the upper, middle, and lower parts of the WM as shown in Figure 3a. The EBSD-IPF map in the upper part occupied almost microstructure by fine grains with spines, indicating acicular ferrite. In the middle part (Figure 3c), fine needle-shaped grains and coarse grains are observed, probably taken near the former γ grain boundary. Finally, in the lower part (Figure 3d), the fine needle-shaped grains are divided by coarse grains, indicating the typical microstructures of acicular ferrite and grain boundary ferrite.

**Figure 4:**
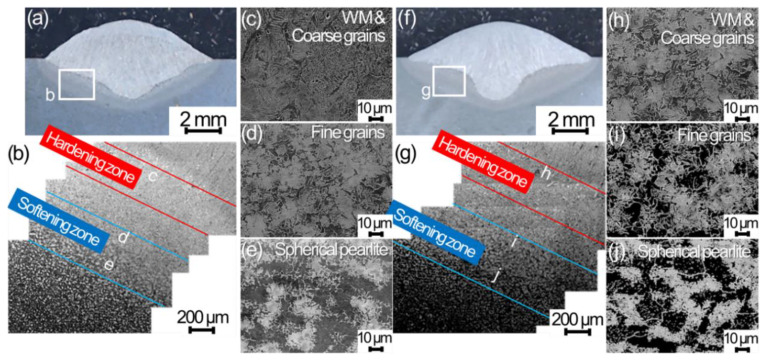
Cross-sectional scanning electron microscope (SEM) images for TP-GMAW specimens at 1.2 m/min (**a**–**c**) w/ and (**f**–**j**) w/o sine vibration (F = 250 Hz, G = 1.2 m/s^2^ and T = 30 s).

The HAZ included coarse grains, mixed grains, fine grains, and spherical pearlite from the boundary between WM and HAZ to BM in order as shown in Figure 4. Cross-sectional scanning electron microscope (SEM) images for TP-GMAW specimens at 1.2 mm/min w/ vibration are shown in Figure 4a–e. The macroscopic SEM image below the concave spot exhibits indistinct gray contrast and white spotted images in order, and the spots grow in a direction away from the boundary between WM and HAZ (Figure 4b). The enlarged images in the positions designated by c, d, and e in Figure 4b are shown in Figure 4c,d,e, respectively. The coarse- and fine-grain zones indicate lath martensite (Figure 4c) and fine ferrite and bainite grains (Figure 4d). The bainite grains exhibit white needle like contrast. Further away from the boundary, cementite with white contrast gathered in a spherical manner, forming spherical pearlite (Figure 4e). The similar microstructure feature was also observed below the WM bottom with less HAZ thickness. Similar macroscopic and microscopic feature also exhibits in HAZ of specimens w/o vibration as shown in Figure 4f–j. Thus, the sine-mode workpiece vibration does change penetration shape, but does not change WM and HAZ macroscopic and microscopic features although HAZ thickness depends on the penetration shape.

**Figure 5:**
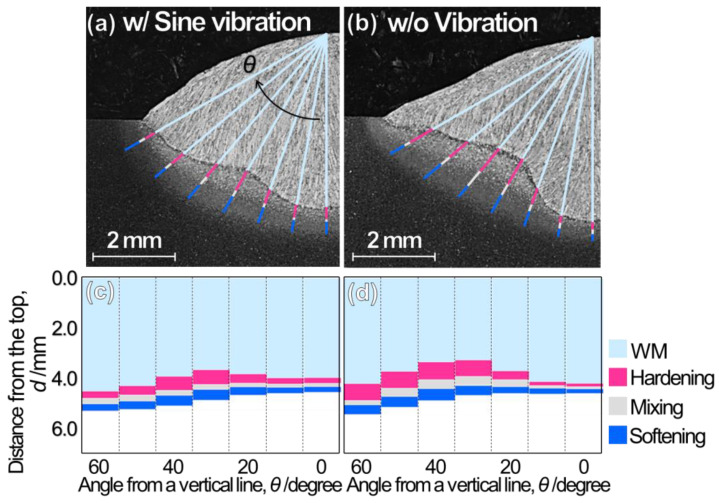
Cross-sectional (**a**,**b**) OM images and (**c**,**d**) bar charts of specific weld metal (WM) and heat-affected zone (HAZ) area thickness for TP-GMAW specimens at 1.2 m/min (**a**,**c**) w/ and (**b**,**d**) w/o sine vibration (F = 250 Hz, G = 1.2 m/s^2^ and T = 30 s).

The sine vibration changes the penetration shape, leading to a change of HAZ thickness distribution below the fusion lines. The HAZ-thickness distribution is shown in Figure 5. The line segments of WM and HAZ including hardening, mixing, and softening areas are drawn on the cross-sectional OM images from the top of WM to the end of the HAZ, clockwise at 10° interval from 0° to 60° (Figure 5a,b). The segment length is displayed by a bar chart in Figure 5c,d. The length of a hardening-area bar reduces for the specimen w/ vibration (Figure 5c) in comparison to that for the specimen w/o vibration (Figure 5d). The length difference between mixing and softening areas is not quite obvious. The WM concave spot reduces for the specimen w/ vibration. The total length of WM and HAZ, with an increase in angle, reduces for the specimens.

**Figure 6:**
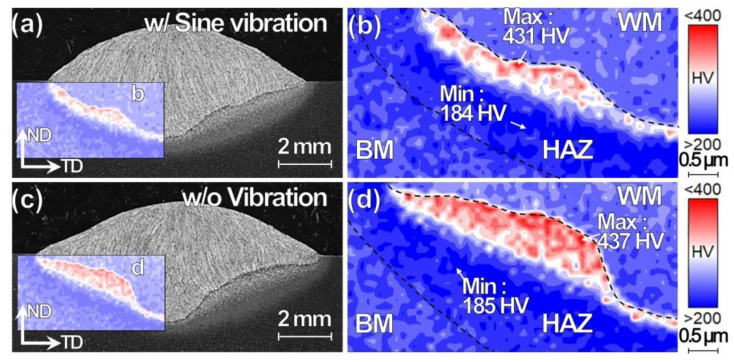
Cross-sectional (**a**,**c**) OM images and (**b**,**d**) hardness mappings for TP-GMAW specimens at 1.2 m/min (**a**,**b**) w/ and (**c**,**d**) w/o sine vibration (F = 250 Hz, G = 1.2 m/s^2^ and T = 30 s).

The weld width reduction and pan-bottom shape formation are probably caused by an increase of molten material flow downward just before solidification is complete. The particular phenomenon is discussed in detail in an earlier investigation on coupled Eulerian Lagrangian (CEL)-based numerical simulation [31]. The HAZ thickness variation affects the local mechanical property. The Vickers hardness tests are carried over the weld area, as shown in the inset figure on the cross-sectional OM images in Figure 6a,c for the specimens w/ and w/o vibration, respectively. Figure 6b,d show the corresponding enlarged hardness mapping. The hardening area formed below the fusion line between WM and HAZ is shown in red. The thin hardening area is distributed uniformly for the specimen w/ vibration; in contrast, the hardening area observed at the concave spot localizes in the specimen w/o vibration. The maximum and minimum hardness values of about 435 and 185 HV, respectively, are similar for both the specimens.

**Figure 7:**
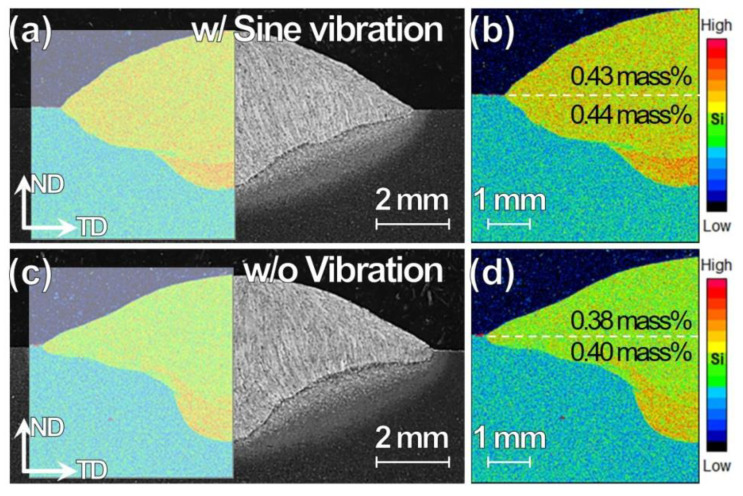
Cross-sectional (**a**,**c**) OM images and (**b**,**d**) electron probe microanalyzer (EPMA)-Si maps for TP-GMAW specimens at 1.2 m/min (**a**,**b**) w/ and (**c**,**d**) w/o sine vibration (F = 250 Hz, G = 1.2 m/s^2^ and T = 30 s).

The sine vibration could affect the molten material flow, and thus the WM dilution is investigated by EPMA-Si mapping where Si is considered as a probe. The Si content is higher in the filler wire than in the BM. The EPMA-Si mapping is conducted in the area, as shown in the inset figure on the cross-sectional OM images in Figure 7a,c and the enlarged mappings are shown in Figure 7b,d for the specimens w/ and w/o vibration, respectively.

The Si segregation is observed at the WM bottom for both the specimens. At the same time, high Si content could be seen uniformly distributed in the WM for the specimen w/ vibration in contrast to the specimen w/o vibration. The average Si contents in the reinforcement area of the weld above and penetration area below the surface of a BM plate are 0.43 and 0.44 mass %, respectively, for the w/ vibration. The corresponding values w/o vibration are 0.38 and 0.40 mass %. It is suggested that the sine vibration assists homogeneous WM dilution.

### 3.2. Variation in Penetration Shape with Welding and Sine Vibration Parameters

**Figure 8:**
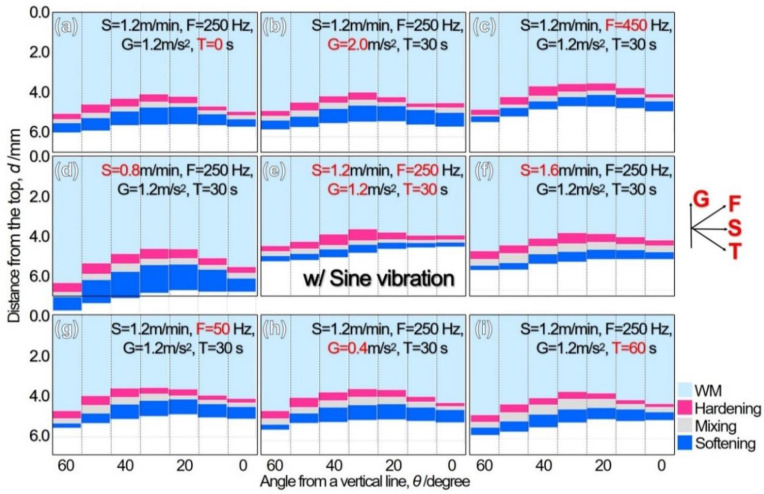
Bar charts of WM and HAZ cross-sectional area thickness for TP-GMAW specimens at specific S, F, G, and T, e.g., (**e**) for the specimen at 1.2 m/mm (F = 250 Hz, G = 1.2 m/s^2^ and T = 30 s).

The line segments of WM and HAZ including hardening, mixing, and softening areas are displayed for the welding parameter (*S*) and sine-vibration parameters (F, G, and T) by a bar chart in Figure 8. The length of a hardening-area bar, as shown in red, is short for almost all the specimens, suggesting that the WM concave spot is reduced by the sine vibration. In contrast, the length of a softening-area bar varies with the parameters. The length increases for the specimen with *S* of 0.8 m/min (Figure 8d) since the slower welding speed increases heat input that also leads to an increase of total HAZ length. Increasing the heat input also increases molten pool volume, leading to deviation from a specific ratio of droplet size to the molten pool size that is related to the resonance phenomenon. With an increase in the welding speed to 1.6 m/min (Figure 8f), the length of a softening-area bar and a total bar do not increase in comparison to the specimen at 1.2 m/min (Figure 8e). In contrast, the minimum length is for the specimen at *F* of 250 Hz, *G* of 1.2 m/s^2^_,_ and *T* of 30 s (Figure 8e) with respect to *F* (Figure 8g,e,c), *G* (Figure 8h,e,b) and *T* (Figure 8a,e,i). Consequently, there is a specific sine-vibration condition that reduces the length of a softening-area bar. The obvious trend change could not be seen in the total HAZ length variation with angle for the specimen at 1.2 m/min.

**Figure 9:**
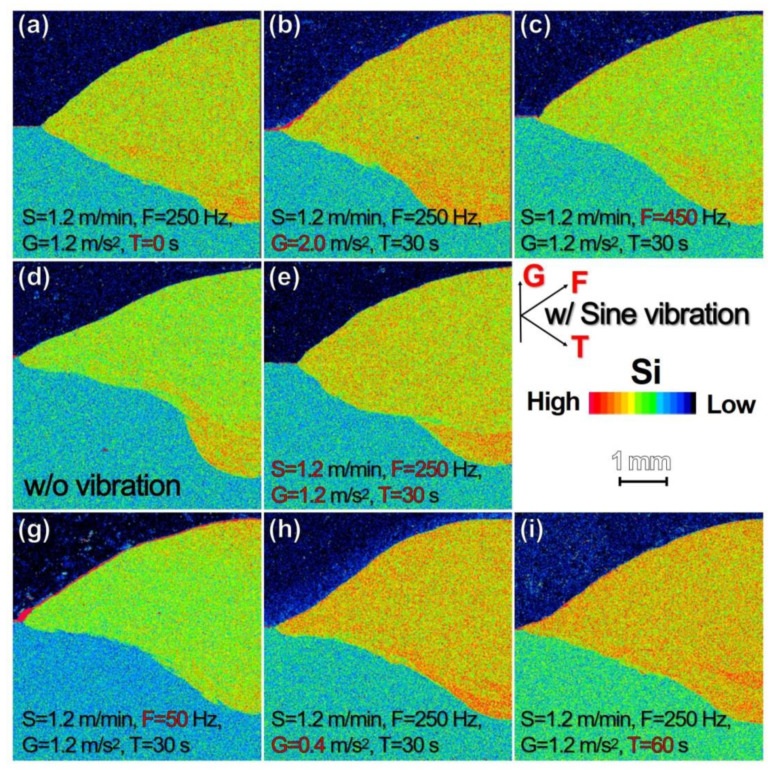
EPMA-Si maps for TP-GMAW specimens at specific S, F, G, and T, e.g., (**e**) for the specimen at 1.2 m/mm (F = 250 Hz, G = 1.2 m/s^2^ and T = 30 s), and for comparison, (**d**) the specimen w/o vibration.

Si distribution in the WM for vibration parameters of F, G and T are summarized in Figure 9, together with that for the specimen w/o vibration (Figure 9d). In comparison to the segregated Si at the WM bottom for the specimen w/o vibration, Si distribution is homogeneous in WM for the specimens w/ vibration for all G (Figure 9h,e,b) and T (Figure 9a,e,i). Similarly, Si distribution is homogeneous for the specimens w/ vibration at F. At the same time, there is relative Si segregation at the WM top and bottom of the specimen at 50 Hz (Figure 9g) and 450 Hz (Figure 9c), respectively. The Si distribution in WM indicates homogeneous mixing of the filler wire and molten BM. Most of the Si in molten material, originally from the filler wire, continues to flow until solidification completes for the specimen w/ vibration. The molten material with more Si would exhibit relatively high temperature, which would allow more of the BM at the concave spot. Consequently, pan-bottom shaped penetration is believed to be created. The amount of molten BM at WM concave spots seems to be the greatest at F of 250 Hz and G of 1.2 m/s^2^ and does not vary with T. The specific sine-vibration at F of 250 Hz, G of 1.2 m/s^2^_,_ and T of 30 s is optimal for changing the penetration shape. The frequency dependence is suggested to be associated with the resonance between the group of molten filer metal droplets and the molten pool.

### 3.3. Vibration Mode Dependence on Penetration Shape Change

**Figure 10:**
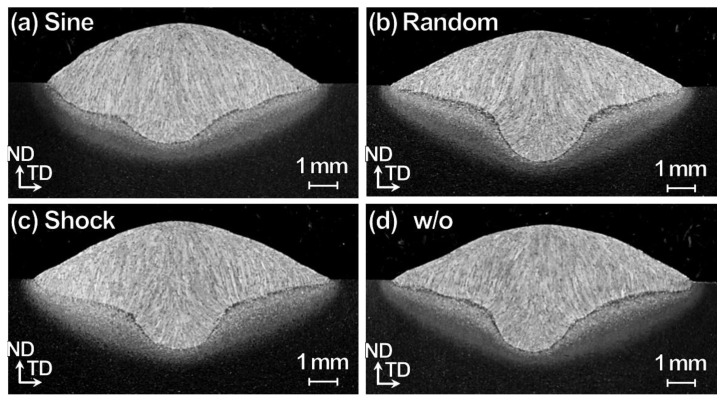
Cross-sectional OM images for TP-GMAW specimens at 1.2 m/min w/ three-mode vibration modes including (**a**) sine, (**b**) random, and (**c**) shock modes (F = 250 Hz, G = 1.2 m/s^2^ and T = 30 s), together with that of (**d**) w/o vibration, for comparison.

Figure 10 shows cross-sectional OM images for specimens at 1.2 m/min with three vibration modes including sine, random, and shock modes (at F = 250 Hz, G = 1.2 m/s^2^ and T = 30 s), together with that of w/o vibration for comparison. The change in weld shape in comparison to the specimen w/o vibration (Figure 10e) is unique for the specimen w/ sine vibration (Figure 10a). A similar change is not observed with random and shock vibrations (Figure 10b,c). Similarly, thinning of HAZ in the specimens w/random and shock vibrations is not noticed. All the specimens exhibited typical microstructural features such as grain size remain similar that are observed in the WM and HAZ of the low carbon steel. The random vibration consisted of many sine vibrations with different amplitudes and frequencies, unlike the shock vibration that consists of a pulse-like waveform, as shown in Figure 1b. The inconsistent amplitude in the random mode and pulse-like displacement in the shock mode do not induce the resonance like that produced by the sine vibration. Consequently, it is necessary to have a continuous vibration of consistent amplitude for the penetration shape change and corresponding HAZ thinning to take place in a specific range of frequency.

**Figure 11:**
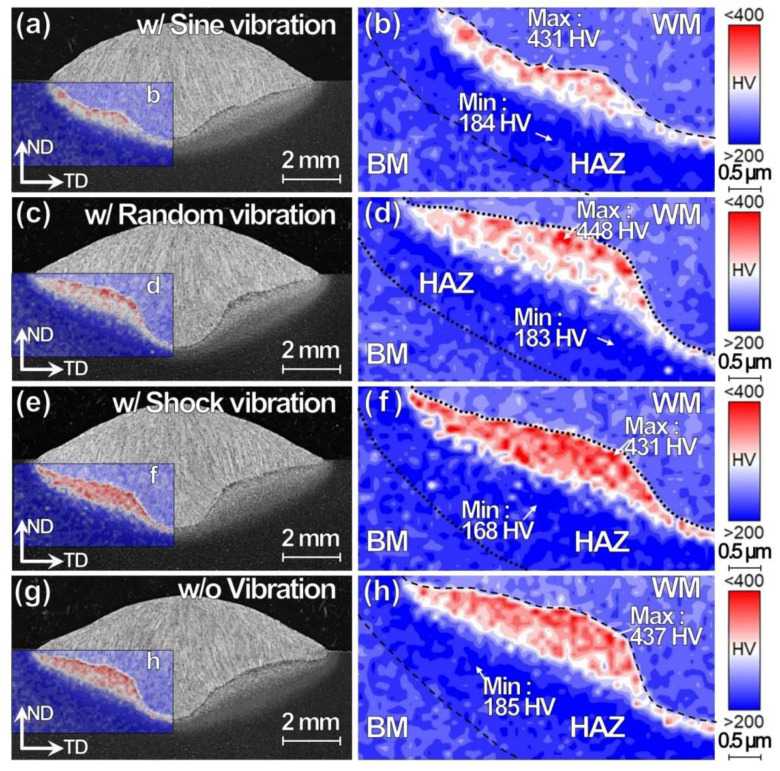
Cross-sectional (**a**,**c**,**e**,**g**) OM images and (**b**,**d**,**f**,**h**) hardness mappings for TP-GMAW specimens at 1.2 m/min w/ three-mode vibrations including (**a**,**b**) sine, (**c**,**d**) random, and (**e**,**f**) shock modes (F = 250 Hz, G = 1.2 m/s^2^ and T = 30 s) and (**g**,**h**) w/o vibration, for comparison.

The penetration shape, HAZ thickness, and hardness variation of the specimen w/ shock and random vibration (Figure 11c–f) remain same as w/o vibration (Figure 11g,h), in contrast to the specimen w/ sine vibration (Figure 11a,b). The hardness variation in the HAZ is attributed to the penetration shape and corresponding HAZ thickness variation. The area with relatively higher hardness, as shown in red in the hardening area, decreases in the random mode (Figure 11d) and increases in the shock mode (Figure 11d), while the maximum values remain similar, in comparison to the specimen w/o vibration. The interrupted random vibration does not provide heat flow favorable for the reduction in the HAZ width, but the reason has not yet been clarified.

**Figure 12:**
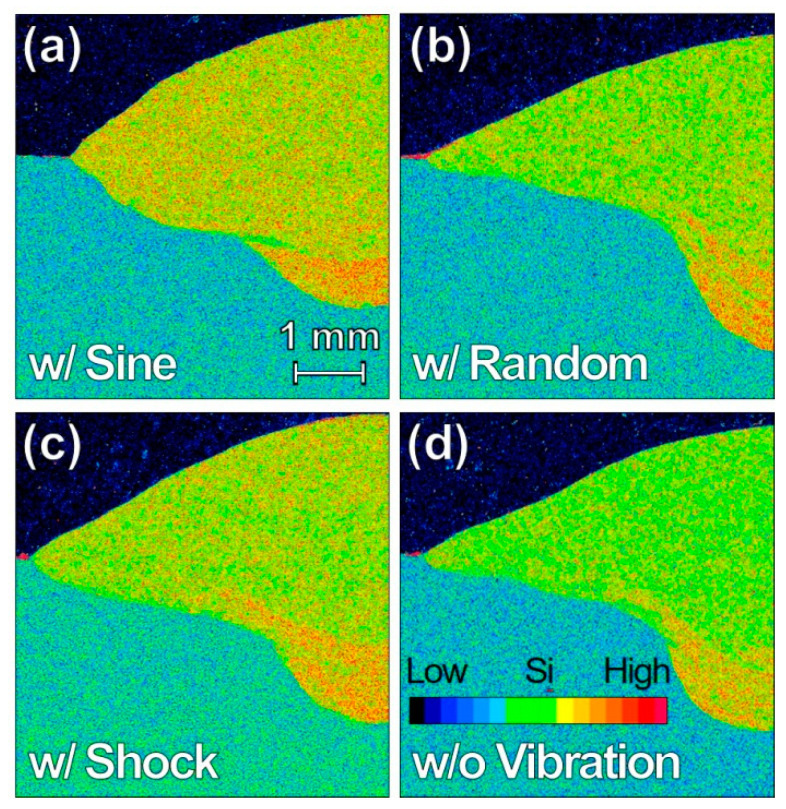
EPMA-Si maps for TP-GMAW specimens at 1.2 m/min w/ (**a**) sine, (**b**) random, (**c**) shock vibration (F = 250 Hz, G = 1.2 m/s^2^ and T = 30 s), and (**d**) w/o vibration.

Given the preceding observations, the WM dilution, as expected, is not affected by random and shock vibrations. Distribution of Si atoms in the WM for the four specimens is summarized in Figure 12. In contrast to the homogeneously distributed Si atoms in WM w/ sine vibration (Figure 12a), the Si atoms are segregated at the penetration bottom of the specimen w/o vibration (Figure 12d). Significant segregation of the Si atoms at the penetration bottom is observed for the specimen w/ random vibration, together with a little more homogeneous distribution in the WM than the specimen w/o vibration. On the other hand, a little less homogeneous distribution of the Si atoms is observed for the specimen w/ shock vibration than the specimen w/ sine vibration, in addition to the segregation at the penetration bottom. The vibration-mode effects on hardness in the HAZ and the WM dilution for the specimens w/ random and shock vibrations are opposite in the influence, which suggests that the proportion of time spent in heat diffusion between the WM and HAZ varies with vibration mode.

### 3.4. Workpiece Vibration-Assisted Change in the Interaction between Two Molten Liquid Groups

The results motivate a new idea about the interaction between two molten liquid groups with higher and lower temperatures in the weld pool. The two molten liquid groups are homogeneously mixed by sine vibration with a designated amplitude along the welding direction, especially at a specific frequency, as mentioned above. The phenomenon is similarly observed in thixotropic materials in the solid–liquid coexistent state [32,33]. Unlike the thixotropic phenomenon, this study engages a liquid–liquid interaction and shape change of high-temperature droplets that impinge on the molten WM. A similar resonance phenomenon would occur in two coexistent liquid groups. It is also conceivable that the viscosity of the solid–liquid coexistence zone near a fusion line plays a vital role in controlling penetration shape associated with the thixotropic phenomenon. The thixotropic phenomenon occurs only in the solid–liquid coexisting state, which is accompanied by shear stress for a certain period in the same direction. The continuous sine vibration produces continuous stress along the fusion line for a certain period and in the same direction. The stress is effective in decreasing viscosity of the solid–liquid coexistence zone at WM concave spots, leading to more molten material flow and melting BM followed by changing penetration shape.

Despite a substantial development in TP-GMAW technology, the results of this investigation show that several investigations are needed to fully understand the vibration welding phenomenon. The investigation provides a scientifically novel idea of weld pool shape control by using vibration during the welding. The future studies need to be pointed towards the grain size characterization, the effect of a broader range of oscillation, and thermo-mechanical phenomenon at the weld interface. Moreover, the weld pool under the influence of the vibration needs to be numerically modelled for visualization of the associated phenomenon.

## 4. Conclusions

The article presents an experimental study on effect of vibration on weld pool formation in tandem pulse gas metal arc welding. The welds produced under the three modes of vibration, namely sine, random and shock vibration are analyzed for the impact on weld pool shape and microstructure due to change in vibration and welding parameters. The investigation draws the following conclusions:

1. The weld metal penetration changes from a finger-shape to a pan-bottom shape by sine-mode workpiece vibration along the welding direction, especially at a specific frequency and a designated amplitude during tandem pulsed gas metal arc welding.

2. The heat-affected zone is homogeneously distributed, and its thickness reduces with sine vibration. The phenomenon is observed at a specific frequency and is associated with the change in penetration shape.

3. The Si atoms supplied by the filler wire distribute homogeneously in the weld metal under the sine vibration at a specific frequency, although Si segregation is observed at the penetration bottom.

4. The homogeneous weld metal dilution and uniformly distributed thin hardening area in heat affected zone are observed only under sine vibration.

5. The changes as caused by sine vibration (1 to 4) are not similarly observed with random and shock vibration. However, the random vibration causes a little change in the weld pool compared to the trivial effect of the shock vibration.

6. Microstructure features of weld metal and heat affected zone do not change by workpiece vibration in sine, shock, and random modes within the range of the investigated parameters.

## Figures and Tables

**Table 1:** The composition of a base metal (BM) and a filler wire.

	C	Si	Mn	P	S	Fe
IS2062-2011	0.211	0.206	0.71	0.015	0.018	Bal.
ER70S-6	0.07–0.15	0.80–1.15	<1.85	<0.025	<0.035	Bal.

**Table 2:** The nine representative condition sets of welding speed and sine-mode vibration parameters in tandem-wire pulsed gas metal arc welding (TP-GMAW).

Sample	S (m/min)	F (Hz)	G (m/s^2^)	T (s)
a	1.2	250	1.2	0 *
b			2.0	30
c		450	1.2	
d	0.8	250		
e	1.2			
f	1.6			
g	1.2	50		
h		250	0.4	
i			1.2	60

* T = 0, stopping vibration upon the welding was finished.
